# Does Intensity Modulated Radiation Therapy (IMRT) prevent additional toxicity of treating the pelvic lymph nodes compared to treatment of the prostate only?

**DOI:** 10.1186/1748-717X-3-3

**Published:** 2008-01-11

**Authors:** Matthias Guckenberger, Kurt Baier, Anne Richter, Dirk Vordermark, Michael Flentje

**Affiliations:** 1 Department of Radiation Oncology, Julius-Maximilians University, Wuerzburg, Germany

## Abstract

**Background:**

To evaluate the risk of rectal, bladder and small bowel toxicity in intensity modulated radiation therapy (IMRT) of the prostate only compared to additional irradiation of the pelvic lymphatic region.

**Methods:**

For ten patients with localized prostate cancer, IMRT plans with a simultaneous integrated boost (SIB) were generated for treatment of the prostate only (plan-PO) and for additional treatment of the pelvic lymph nodes (plan-WP). In plan-PO, doses of 60 Gy and 74 Gy (33 fractions) were prescribed to the seminal vesicles and to the prostate, respectively. Three plans-WP were generated with prescription doses of 46 Gy, 50.4 Gy and 54 Gy to the pelvic target volume; doses to the prostate and seminal vesicles were identical to plan-PO. The risk of rectal, bladder and small bowel toxicity was estimated based on NTCP calculations.

**Results:**

Doses to the prostate were not significantly different between plan-PO and plan-WP and doses to the pelvic lymph nodes were as planned. Plan-WP resulted in increased doses to the rectum in the low-dose region ≤ 30 Gy, only, no difference was observed in the mid and high-dose region. Normal tissue complication probability (NTCP) for late rectal toxicity ranged between 5% and 8% with no significant difference between plan-PO and plan-WP. NTCP for late bladder toxicity was less than 1% for both plan-PO and plan-WP. The risk of small bowel toxicity was moderately increased for plan-WP.

**Discussion:**

This retrospective planning study predicted similar risks of rectal, bladder and small bowel toxicity for IMRT treatment of the prostate only and for additional treatment of the pelvic lymph nodes.

## Background

In 2003 the randomized phase III Radiation Therapy Oncology Group (RTOG) trial 94-13 showed improvement of progression free survival (PFS) for whole pelvis (WP) radiotherapy compared to treatment of the prostate only (PO) [1]. Patients with elevated prostate-specific antigen (PSA) ≤100 ng/ml and an estimated risk of lymph node involvement >15% based on pre-treatment PSA value and Gleason score [2] were randomized between PO vs. WP radiotherapy and neoadjuvant and concurrent vs. adjuvant androgen depression: 4-year PFS was 54% and 47% in the WP and PO treatment arms, respectively. However, this difference was smaller based on an updated analysis from 2007 [3]. Consequently, radiotherapy treatment of the pelvic lymphatics for patients with localized prostate cancer remains controversy.

With conventional or three-dimensional conformal radiotherapy (3D-CRT), the treatment of the pelvic lymphatics ultimately results in increased doses to the organs-at-risk (OAR) rectum, bladder and small bowel compared to treatment of PO. Whereas no correlation between field size and late genitourinary toxicity was seen in the RTOG 94-13 trial, a positive correlation was observed for late Grade 3+ gastrointestinal toxicity: larger field sizes with larger volumes of the rectum within the high-dose region resulted in increased rates of toxicity [1]. Updated results showed only a higher rate of late grade 3+ gastrointestinal toxicity for men treated with whole-pelvic RT with neaoadjvant RT (5%) compared to patients treated with whole pelvis RT and adjuvant androgen deprivation therapy (2%) and prostate only (1% with androgen deprivation therapy and 2% without androgen deprivation therapy) [3]. The authors suggest there may be an unexpected relationship between the timing of androgen deprivation and whole pelvis radiotherapy.

The close proximity of the prostate and the pelvic lymphatics to the bladder, rectum and small bowel encouraged the use of intensity-modulated radiotherapy (IMRT) for prostate cancer [4,5]. Multiple planning studies demonstrated more conformal dose distributions and decreased doses especially to the rectum for IMRT compared with 3D-CRT in treatment of PO [6-8]. Early clinical results confirmed the potential of IMRT with low rates of toxicity despite escalated treatment doses to the prostate [9-12]. Analogously, planning studies reported reduced doses to the rectum, small bowel and bladder for IMRT compared with 3D-CRT in treatment of WP [13-15].

Though planning studies proved the advantage of IMRT compared to 3D-CRT in treatment of PO as well as treatment of the WP, it is not possible to estimate the additional risk of IMRT treatment of the pelvic lymphatics compared to IMRT treatment of the PO. Differences in the design of the planning and clinical studies (target volume definition, treatment planning, treatment machine, single fraction dose, total dose) make a comparison difficult. We conducted this intra-individual planning study to evaluate, whether there exists a risk of increased toxicity in treatment of the pelvic lymph nodes in the IMRT era.

## Methods

This retrospective planning study included ten consecutive patients treated for localized prostate cancer at the Department of Radiation Oncology of the University of Wuerzburg, Germany, between August 2006 and November 2006. The target volume in real patient treatment had been PO and WP in five and five patients, respectively.

A spiral planning CT scan was acquired in supine position. Slice thickness was 3 mm. Additionally, a planning MRI was acquired for all patients; slice thickness was identical to the planning CT with 3 mm. Patients were advised to have an empty bowel and a full bladder at the time of treatment planning and during the treatment.

ADAC Pinnacle treatment planning system (TPS) v8.1s (Philips/ADAC, Milpitas, CA, USA) was used for registration of the planning CT and MRI, for target volume definition, treatment planning and plan evaluation.

**Figure 1:**
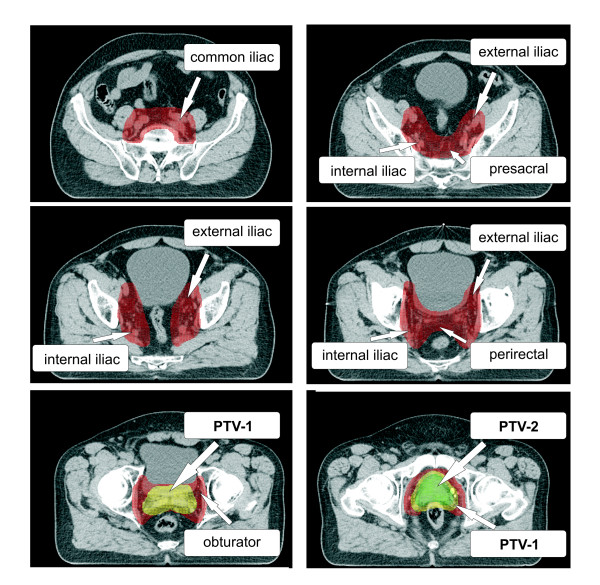
Composition of the target volumes PTV-1, PTV-2 and PTV-LAG.

The prostate and seminal vesicles were contoured in the planning CT and the planning MRI and the sum of both structures was defined as the clinical target volume (CTV). The CTV-1 was the prostate including seminal vesicles and the CTV-2 was the prostate and base of the seminal vesicles. The CTV-1 was expanded with a 3D margin of 10 mm resulting in the planning target volume 1 (PTV-1), to posterior the margin was limited to 7 mm. A 3D margin of 5 mm was added to the CTV-2 resulting in the PTV-2, overlap with the rectum was not allowed. The pelvic lymphatic drainage comprised the obturator, peri-rectal, internal iliac, proximal external iliac and common iliac lymph nodes up to L5/S1 (Fig. 1). The delineation of the PTV-LAG was based on the large pelvic vessels rather than the bony anatomy as suggested by Shih *et al.*[16]. Definition of target volumes is summarized in Table 1. The bladder, rectum, small bowel and femoral heads were delineated as OARs. The bladder and rectum were contoured as solid rectal volume (RV) and solid bladder volume (BV) as well as rectal wall (RW) and bladder wall (BW).

**Table 1:** Definition of target volumes

**Target volumes**	**CTV definition**	**PTV definition**
PTV-1	Prostate and seminal vesicles	+ 10 mm uniform margin, 7 mm to posterior
PTV-2	Prostate and base seminal vesicles	+ 5 mm uniform margin, no overlap with rectum
PTV-LAG	Obturator, peri-rectal, internal iliac, proximal external iliac, common iliac lymph nodes up to L5/S1	+ 10 mm margin around large pelvic vessels

### IMRT treatment planning

Treatment was planned for an Elekta Synergy S linac (Elekta, Crawley, England) equipped with the beam modulator with 4 mm leaf width and step-and-shoot IMRT technique. The isocentre was placed in the geometrical centre of the PTV-1 for treatment of PO (plan-PO) and in the geometrical centre of PTV-LAG for treatment of the WP (plan-WP). Seven beams were generated with gantry angles of 0°, 51°, 103°, 155°, 206°, 258° and 309°. Photon energy was 10 MV.

**Figure 2:**
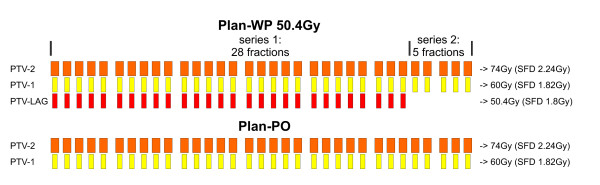
Schematic protocols of plan-PO and plan-WP (dose prescription of 50.4 Gy to PTV-LAG).

For both plan-PO and plan-WP, IMRT class-solutions with a simultaneous-integrated-boost (SIB) were developed. Schematic protocols of plan-PO and plan-WP are shown in Figure 2. In plan-PO, the prescription dose [the minimum dose that is delivered to 95% of the target volume (D95)] was 60 Gy to the PTV-1 and 74 Gy to the PTV-2 in 33 fractions. This resulted in single fraction doses (SFD) of 1.82 Gy to PTV-1 and 2.24 Gy to PTV-2. Based on an α/β ratio of 1.5 Gy, 3 Gy or 10 Gy [17,18] for the prostate this fractionation schema equates a 1.8 Gy equivalent dose of 83.9 Gy, 80.7 Gy or 76.8 Gy, respectively.

For treatment of the WP, three plans were generated with prescription doses of 46 Gy, 50.4 Gy and 54 Gy to the PTV-LAG; prescription doses to PTV-1 and PTV-2 were identical to plan-PO. Because of the large difference in the total dose between PTV-LAG and PTV-2, one single IMRT plan with SIB was not possible: the differences in the SFD would be too large. Therefore, plan-WP was split into two IMRT series, each with a SIB. The first series was an IMRT plan with 25, 28 and 30 fractions for a total dose of 46 Gy, 50.4 Gy and 54 Gy to the PTV-LAG, respectively. Using the SIB concept, the SFD to the PTV-1 and the PTV-LAG was between 1.80 Gy and 1.84 Gy, respectively; SFD to the PTV-2 was 2.24 Gy. The PTV-LAG was excluded from the second IMRT series and the IMRT optimization objectives were identical to plan-PO. However, only eight, five and three fractions were prescribed for the plans with doses of 46 Gy, 50.4 Gy and 54 Gy to PTV-LAG in the first series, respectively. This resulted in total doses of 60 Gy and 74 Gy to the PTV-1 and the PTV-2, respectively. Consequently, the single fraction dose and total dose to the prostate were identical between treatment of the PO and WP: plan-PO and plan-WP differed in the treatment of the pelvic lymph nodes, only.

Optimization objectives for plan-PO and plan-WP are listed in Table 2 and 3. The minimum segment area was 4 cm^2 ^and the minimum number of monitor units was 4 for one segment. The maximum number of segments was 30 for plan-PO and 50 for the first series in plan-WP. Direct-machine-parameter-optimization (DMPO) was used with sequencing simultaneously to the inverse optimization process.

**Table 2:** Objectives for IMRT treatment planning of plan-PO

**Target Volumes**			
	**D min**	**D 95**	**D max**

**PTV-1**	56 Gy	60 Gy	
**PTV-2**	70 Gy	74 Gy	80 Gy
**Organs at risk**			

**PTV-1 sine PTV-2**	Max 74 Gy to 5%	Max 66 Gy to 50%	D_max _78 Gy
**Help contour ring 1.5 cm**	Max 60 Gy to 10%	Max 45 Gy to 50%	
**Bladder (BV)**	Max 70 Gy to 5%	Max 50 Gy to 20%	Max 30 Gy to 30%
**Rectal volume sine PTV**	Max 60 Gy to 5%	Max 40 Gy to 20%	Max 20 Gy to 40%
**Seminal vesicles**	Max 65 Gy to 20%		

"PTV-1 sine PTV-2" is this proportion of the PTV-1 that does not overlap with PTV-2 – it is listed in organs-at-risk to limit doses >74 Gy to PTV-2. "Rectal volume sine PTV" is the portion of the rectum located outside PTV-1. These objectives were adjusted for the patient's anatomy to achieve optimal results. "Help contour ring" is a 15 mm wide ring shaped contour around the PTV-1 to confine high and mid doses to the target volume.

**Table 3:** Objectives for IMRT treatment planning of plan-WP with a prescribed dose of 50.4 Gy to PTV-LAG

**Target Volumes**			
	**D min**	**D 95**	**D max**

**PTV-1**	47.5 Gy	50.9 Gy	
**PTV-2**	59.4 Gy	62.8 Gy	68 Gy
**PTV-LAG**	47.5 Gy	50.4 Gy	
**Organs at risk**			

**PTV-1 sine PTV-2**	Max 62.8 Gy to 5%	Max 56 Gy to 50%	D max 66.2 Gy
**Help contour ring 1 cm**	Max 50 Gy to 15%	Max 40 Gy to 50%	D max 54 Gy
**Help contour ring 2–3 cm**	Max 30 Gy to 25%	Max 20 Gy to 60%	D max 40 Gy
**Bladder (BV)**	Max 60 Gy to 5%	Max 45 Gy to 20%	Max 30 Gy to 50%
**Rectal volume sine PTV**	Max 50 Gy to 5%	Max 35 Gy to 20%	Max 25 Gy to 70%
**Seminal vesicles**	Max 55 Gy to 20%		

"Help contour ring 1 cm" was a 10 mm wide ring shaped contour around the PTV-1 and PTV-LAG. "Help contour ring 2–3 cm" was a 20 mm wide ring shaped contour around "Help contour ring 1 cm".

After plan generation, series one and series two of plan-WP were accumulated and compared with plan-PO. All plans were normalized to a mean dose of 76.5 Gy to the PTV-2. Dose-volume histograms (DVH) were calculated for target volumes and OARs. Vx was defined as the volume that is exposed to at least xGy. For the rectum (RV and RW), the bladder (BV and BW) and the small bowel normal-tissue complication probabilities (NTCP) were calculated using the relative seriality model described by Källman *et al.*[19]. Radiation tolerance data from Emami *et al*. [20] were fitted to the relative seriality model [21]: parameters for NTCP calculation are listed in Table 4. For the small bowel a secondary set of tolerance data [22] based on clinical results of small bowel toxicity published by Letschert *et al.*[23] was applied.

**Table 4:** Radiation tolerance data for calculation of NTCP using the relative seriality model

	**D50**	**Gamma**	**α/β ratio**	**seriality**
**Rectum**	80 Gy	2.2	3 Gy	1.5
**Bladder**	80 Gy	3	3 Gy	0.18
**Small bowel I**	53.6 Gy	2.3	3 Gy	1.5
**Small bowel II**	62 Gy	2.1	3 Gy	0.14

Plan-PO and plan-WP were compared using student's t-test. For statistical analysis Statistica 6.0 (Statsoft, Tulsa, USA) was utilized. Differences were considered significant for p < 0.05.

## Results

### Dose to the target volumes

**Figure 3:**
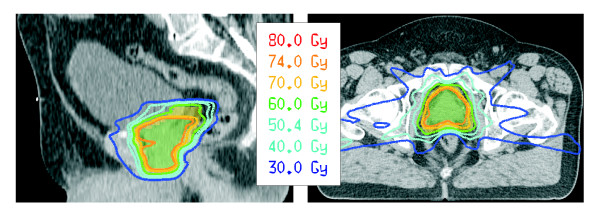
Representative dose distributions for plan-PO.

**Figure 4:**
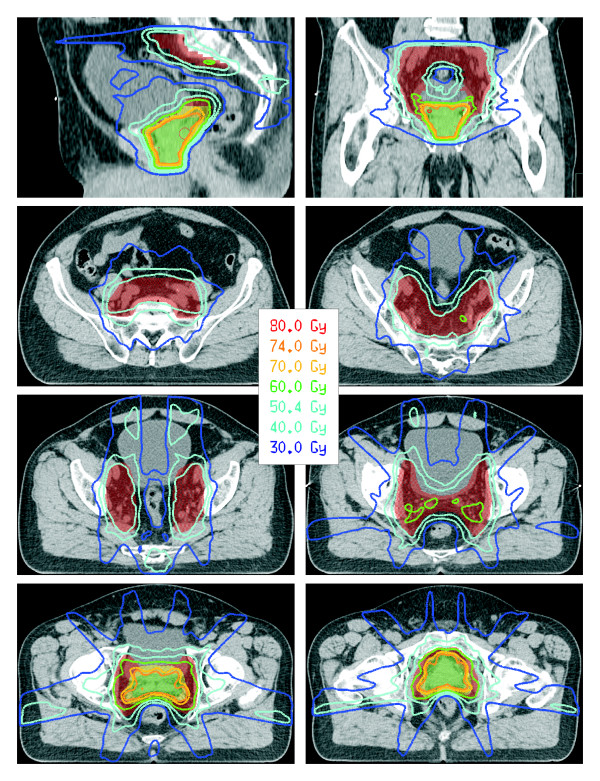
Representative dose distributions for plan-WP.

Representative dose distributions for plan-PO and plan-WP are shown in Figure 3 and Figure 4, respectively. After normalization of all plans to a mean dose of 76.5 Gy to PTV-2, the D95 dose to the PTV-1 was higher than the prescribed dose of 60 Gy and the D95 dose to the PTV-2 was lower than 74 Gy (Table 5). This is explained by the small distance between the structures PTV-1 and PTV-2 in posterior direction where a dose gradient of 14 Gy was not possible. Plan-PO resulted in slightly higher D95 doses to PTV-1 and PTV-2 compared to plan-WP; these differences were in the range of 0.5 Gy or less and not statistically significant. In plan-WP the D95 doses to the PTV-LAG were close to the prescribed doses of 46 Gy, 50.4 Gy and 54 Gy (Table 5).

**Table 5:** Doses to the target volumes in plan-PO and plan-WP

	**PTV-2 D95 (Gy)**	**PTV-1 D95 (Gy)**	**PTV-LAG D95 (Gy)**
**Plan-PO**	72.5 ± 0.5	62 ± 2.0	
**Plan-WP 46 Gy**	72.3 ± 0.6	61.8 ± 1.8	45.6 ± 0.5
**Plan-WP 50.4 Gy**	72 ± 0.8	61.6 ± 1.6	49.7 ± 0.7
**Plan-WP 54 Gy**	71.9 ± 0.9	61.5 ± 1.6	53.2 ± 0.9

Doses to the target volumes PTV-1, PTV-2 and PTV-LAG in plan-PO and plan-WP. Averaged values for all ten patients.

### Comparison of plan-PO and plan-WP 50.4 Gy

**Figure 5:**
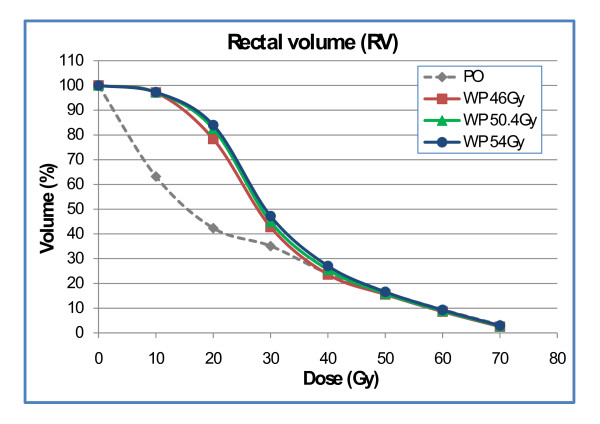
Dose-volume histogram of the rectal volume for plan-PO and plan-WP.

With the analysis based on the rectum as a solid organ, differences between plan-WP and plan-PO were significant in the low dose region (p < 0.001), only: plan-WP resulted in increased rectal volumes exposed to 10 Gy (V10: 97% ± 3% vs. 62% ± 14%) and exposed to 20 Gy (V20: 83% ± 13% vs. 42% ± 12%). The difference between plan-WP and plan-PO for V30 did not reach statistical significance (45% ± 15% vs. 35% ± 12%) (p = 0.14). Volumes of the RV exposed to mid and high doses of 40 Gy to 70 Gy were almost identical (Fig. 5).

**Figure 6:**
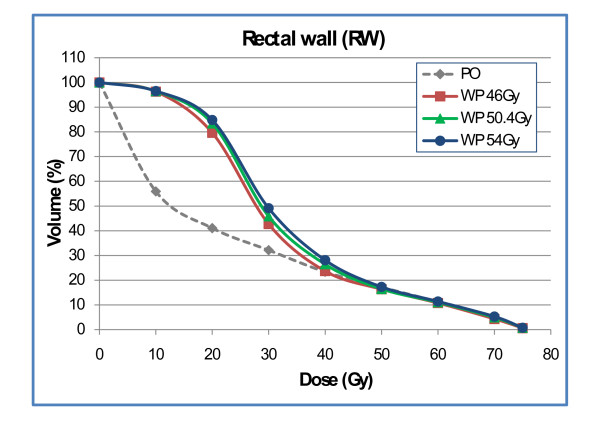
Dose-volume histogram of the rectal wall for plan-PO and plan-WP.

Results based on the RW were similar: treatment of the pelvic lymphatics increased the dose to the rectal wall only in the 10 Gy to 30 Gy region with no difference in the mid and high dose region (Fig. 6). NTCP calculations of late rectal toxicity confirmed data from DVH analysis: no difference between plan-PO and plan-WP was observed (Table 6). The risk for late rectal toxicity was 5% to 6% based on the RV and 7% to 8% based on the RW.

**Table 6:** NTCP for late rectal toxicity in plan-PO and plan-WP

	**NTCP for late rectal toxicity (%)**	
	
	**Rectal volume (RV)**	**Rectal wall (RW)**
**Plan-PO**	5.7% ± 1.8%	7.5% ± 2.2%
**Plan-WP 46 Gy**	5.8% ± 1.3%	7.6% ± 1.5%
**Plan-WP 50.4 Gy**	5.9% ± 1.1%	7.8% ± 1.1%
**Plan-WP 54 Gy**	6.1% ± 1.2%	8.1% ± 1.1%

Normal tissue complication probability (NTCP) for late rectal toxicity in plan-PO and plan-WP. Averaged values for all ten patients.

**Figure 7:**
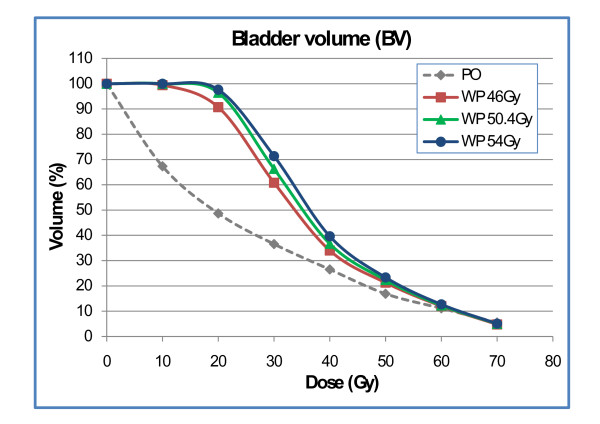
Dose-volume histogram of the bladder volume for plan-PO and plan-WP.

**Figure 8:**
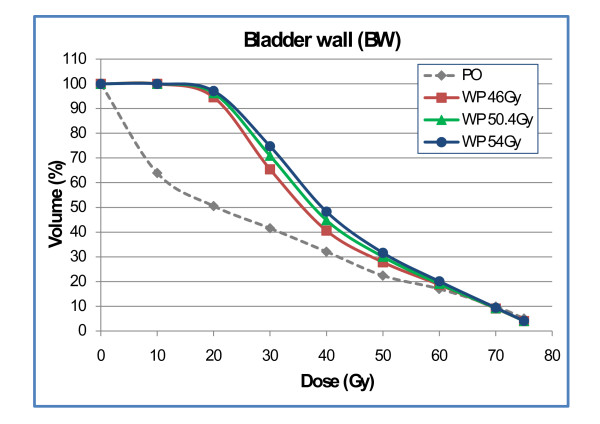
Dose-volume histogram of the bladder wall for plan-PO and plan-WP.

Doses to the BV were increased for plan-WP compared to plan-PO in the region of V10 to V40; no significant difference was observed for V50 to V70 (Fig. 7). At the 50 Gy dose level, the prescription dose to the PTV-LAG, the difference between plan-WP and plan-PO did not reach statistical significance (22% ± 9% vs. 17% ± 7%). Results for delineation of the BW were more pronounced. Plan-WP resulted in greater volumes of the BW exposed to low and mid doses from 10 Gy to 50 Gy: the difference at the 50 Gy dose level was significant with 30% ± 8% vs. 23% ± 6% (Fig. 8). These increased doses to the bladder in the low and mid dose region did not transfer into higher risk of late bladder toxicity: NTCP calculations resulted in risk values of <1% for both plan-PO and plan-WP.

No small bowel was exposed to doses of 36 Gy or higher in plan-PO. In plan-WP the sparing of the small bowel was successful with 7 ccm ± 8 ccm and 27 ccm ± 27 ccm of small bowel exposed to 45 Gy and 36 Gy, respectively. NTCP calculations for small bowel toxicity showed no risk for plan-PO. Based on tolerance data I, plan-WP increased the risk of small bowel toxicity to 2.3% ± 2.5%, maximum 6.4% in one single patient; based on tolerance data II, the risk of small bowel toxicity was 0%. Detailed results are shown in Table 7.

**Table 7:** DVH analysis and NTCP for the small bowel in plan-PO and plan-WP

	**Small bowel**			
	
	**V36 (ccm)**	**V45 (ccm)**	**NTCP (%) I**	**NTCP (%) II**
**Plan-PO**	0	0	0%	0%
**Plan-WP 46 Gy**	13 ± 16	3 ± 5	0.8% ± 1%	0%
**Plan-WP 50.4 Gy**	27 ± 27	7 ± 8	2.3% ± 2.5%	0%
**Plan-WP 54 Gy**	36 ± 38	10 ± 11	3.2% ± 3.5%	0%

DVH analysis and normal tissue complication probability (NTCP) for the small bowel in plan-PO and plan-WP. Averaged values for all ten patients.

### Different dose prescriptions to PTV-LAG

Increasing the prescription dose to PTV-LAG from 46 Gy to 50.4 Gy and to 54 Gy resulted in mild increased doses to the OARs rectum, bladder and small bowel. Similar to previous results, the influence of the prescribed dose to the PTV-LAG on the dose to the RW and BW was lager than the influence on the dose to the RV and BV. The maximum effect was observed at the V30 dose level: a dose of 46 Gy, 50.4 Gy and 54 Gy to PTV-LAG resulted in V30 values of 43% ± 15%, 46% ± 15% and 49% ± 15% to the RW (n.s.), respectively. For the BW, V30 values of 66% ± 8%, 71% ± 8% and 75% ± 8% were calculated (n.s.). At the V50 dose level, no difference was observed for the rectum; this was valid for delineation of the RV and of the RW. The partial volume of the BW exposed to 50 Gy was 28% ± 8%, 30% ± 8% and 32% ± 8% for plan-WP with prescription dose of 46 Gy, 50.5 Gy and 54 Gy, respectively.

NTCP calculations for late rectal toxicity showed only small differences between plans with prescription doses ranging between 46 Gy and 54 Gy to the PTV-LAG (Table 6). An escalation of the dose to the PTV-LAG from 46 Gy to 54 Gy increased the risk of rectal toxicity from 5% ± 1% to 6% ± 1% based on the RV and from 7% ± 2% to 8% ± 1% based on the RW (n.s.). The risk of late bladder toxicity was <1% regardless of the dose to the PTV-LAG.

Doses to the small bowel were modestly increased by escalation of the dose to PTV-LAG (Table 7). Based on tolerance data I, higher treatment doses to the PTV-LAG increased the risk of small bowel toxicity, whereas no risk of small bowel toxicity was calculated based on tolerance data II.

## Discussion

This retrospective planning study indicates that treatment of the pelvic lymph nodes does not add significant rectal, bladder or small bowel toxicity compared to treatment of the prostate only: IMRT treatment planning enabled highly conformal dose distributions with excellent sparing of these OARs.

Treatment of the pelvic lymph nodes increased doses to the rectum in the low-dose region up to 30 Gy, only. These results were similar for delineation of the rectum as solid organ and for delineation of the rectal wall. This low dose region is not considered to be of major relevance for acute and late toxicity. Multiple studies reported a dose-volume relationship for rectal toxicity in treatment of the prostate cancer. All studies concluded that the high and mid dose region is most predictive for rectal toxicity [24-31], whereas no correlation between rectal toxicity and exposure with doses of less than 40 Gy was observed. NTCP calculations in our study are concordant with no significant differences between treatment of PO and WP: the risk of late rectal toxicity was in the range of 5% to 8% for prescription doses of 46 Gy, 50.4 Gy or 54 Gy to the pelvic lymphatics.

Treatment of the pelvic lymphatics influenced the dose distribution to the bladder significantly more compared to the dose to the rectum. This is explained by overlap of the lymphatic target volume with the bladder superior to the prostate and seminal vesicles whereas such overlap with the rectum was completely avoided. Plan-WP increased proportions of the bladder wall exposed to low doses (10 Gy to 30 Gy) and to mid doses (40 Gy to 50 Gy); no difference in the high dose region was observed. Despite these differences in the DVH, the risk of late bladder toxicity was less than 1% for both treatment of PO and of WP based on NTCP calculations.

The dose-volume response of the urinary bladder is less well understood compared to the rectum. Cheung et al. investigated dose volume factors associated with an increased risk of late urinary toxicity [32]; all patients had been treated with 78 Gy in the MD Anderson dose escalation study [33]. The hottest volume (hotspot) model was found to be the best-fitting model. The analysis from Peeters et al. was based on the Dutch dose escalation trial [34]. Acute genitourinary toxicity grade 2 or worse was correlated with the absolute bladder surfaces irradiated to ≥40 Gy, 45 Gy, and 65 Gy. The RTOG 94-06 data was analysed by Valicenti *et al.*[35]. The percent of the bladder receiving doses higher than the reference dose (68.4 Gy, 73.8 Gy, or 79.2 Gy) was a significant predictor of acute GU effects. Karlsdóttir et al. reported a retrospective single institution experience with a prescribed dose of 70 Gy to the prostate [36]. Contrary to previous results the toxicity was correlated with rather low doses to the bladder: the fractional bladder volume receiving more than 14 Gy-27 Gy showed the statistically strongest correlation with acute GU toxicity. Nuyttens et al. did not find a dose-response relationship for urinary toxicity after 3D-CRT treatment of prostate cancer with 72 Gy – 80 Gy [37].

For the small bowel, the TD5/5, the dose at which 5% of patients will experience toxicity within 5 years, has been shown to be approximately 45 Gy to 50 Gy [23,38]. IMRT treatment planning resulted in sufficient sparing of the small bowel. No risk of small bowel toxicity is expected after treatment of PO. Based on Emami tolerance data [20], the risk of small bowel toxicity was moderately increased for treatment of the pelvic lymphatic region whereas no risk was calculated using updated tolerance data [22] based on Letschert et al. [23].

These low risk estimations for rectal, bladder and small bowel toxicity are remarkable in consideration of the escalated dose to the prostate: a D95 dose of 74 Gy was prescribed in 33 fractions. Based on an α/β ratio of 1.5 Gy, 3 Gy or 10 Gy for the prostate this hypo-fractionated schema equates a 1.8 Gy equivalent dose of 83.9 Gy, 80.7 Gy or 76.8 Gy, respectively. It is discussed controversially whether irradiation of the pelvic lymph nodes is required in dose escalated treatment of the prostate [39,40] or with long term hormonal therapy [41]. Nevertheless, data from this study indicate that the IMRT technique enables dose escalation to the prostate and simultaneous treatment of the pelvic lymph nodes without increased risk of toxicity.

An integrated boost concept for the prostate while treating the pelvic lymphatic region might be an issue of concern. Motion of the prostate independently from the bony anatomy is well known [42,43] and the clinical significance of these internal set-up errors has been proven [44,45]. Correction of such internal set-up errors by means of image-guided treatment might decrease the coverage of the lymphatic target volume as the pelvic lymph nodes are not expected to move synchronously with the prostate. Hsu et al. investigated this issue, recently [46]: correction of set-up errors was simulated by shifting the original isocenter of the IMRT plan. The influence of these shifts on the dose to the pelvic target volume was reported to be small; coverage of the pelvic target volume was decreased by less than 1%. However, the small number of five patients certainly requires further investigation of this issue.

One limitation of this study is the fact that the calculations are based on one single planning CT study. All patients were advised to empty their rectum about 1.5 hours prior to acquisition of the planning CT and prior to every treatment fraction. The bladder was kept full by drinking of about 500 ccm in that 1.5 hours interval. This procedure has been chosen as an empty rectum was proven to be most representative for the entire course of treatment [47,48] resulting in lower variability of the prostate position, improved target coverage and higher rates of local control [44]. Our results of doses to the rectum are consequently considered to be representative for the total time of treatment. Treatment with a full bladder has been standard protocol as this was shown to result in lower rates of bladder toxicity [49]. However, several studies reported a time trend to decreased bladder filling during treatment if the planning was based on a full bladder [47,50,51]: a motion of the superior and anterior bladder wall towards inferior into areas of higher doses might be the consequence. Additionally, a synchronous motion of small bowel might increase the risk of toxicity compared to results of this study. As all patients at our department are treated with soft-tissue image-guidance using a kV cone-beam CT [52,53], we are currently investigating this issue.

The target volume, which covers the lymphatic drainage of the prostate adequately, has been discussed, intensively. Compared to historical data, surgical series reported a significantly higher incidence of lymphatic disease if the surgical dissection was extended beyond the obturator and external iliac lymph nodes [54,55]. The sentinel lymph node concept has been adapted from breast cancer and malignant melanoma and surgical series showed promising early results [56,57]. Most important for radiotherapy was the finding that there was no uniform pattern of lymphatic drainage. Ganswindt et al. integrated this sentinel lymph node concept into radiotherapy treatment [58,59]. After intraprostatic injection of 250 MBq ^99m^Tc-Nanocoll and SPECT imaging, sentinel lymph nodes outside the standard target volume were detected in 17 of 25 patients. IMRT treatment planning ensured adequate coverage of these complex shaped lymphatic target volumes with significantly lower doses to the rectum and bladder compared to 3D-CRT. As suggested by Shih et al. the definition of the lymphatic target volume was based on the major pelvic vasculature in our study, not on bony landmarks [16]; adequate coverage of the lymphatic drainage of the prostate is therefore expected.

## Conclusion

This retrospective planning study showed similar risk of rectal, bladder and small bowel toxicity for IMRT treatment of the prostate only and for additional irradiation of the pelvic lymph nodes. The decision whether to treat the lymphatic drainage or not should therefore be based on loco-regional control data rather than toxicity data of trials using conventional techniques or 3D-CRT. Clinical data will be necessary to prove this hypothesis.

## Competing interests

The author(s) declare that they have no competing interests.

## Authors' contributions

All authors read and approved the final manuscript.

MG designed the study and the analysis, generated the treatment plans, performed the analysis drafted and revised the manuscript.

KB participated in the study design and revised the manuscript.

AR participated in the generation of IMRT plans and revised the manuscript.

DV participated in the study design and revised the manuscript.

MF participated in the study design and revised the manuscript.
